# Transcriptome and Methylome Profiling in Rat Skeletal Muscle: Impact of Post-Weaning Protein Restriction

**DOI:** 10.3390/ijms232415771

**Published:** 2022-12-12

**Authors:** Sihui Ma, Emi Hasegawa, Yuji Nakai, Huijuan Jia, Hisanori Kato

**Affiliations:** 1 Health Nutrition, Graduate School of Agricultural and Life Sciences, The University of Tokyo, Tokyo 1138657, Japan; 2 Institute of Regional Innovation, Hirosaki University, 2-2-1 Yanagawa, Aomori-shi 0380012, Japan

**Keywords:** methylome, post-weaning, protein restriction, skeletal muscle, transcriptome

## Abstract

Skeletal muscle is programmable, and early-life nutritional stimuli may form epigenetic memory in the skeletal muscle, thus impacting adult muscle function, aging, and longevity. In the present study, we designed a one-month protein restriction model using post-weaning rats, followed by a two-month rebound feeding, to investigate how early-life protein restriction affects overall body growth and muscle development and whether these influences could be corrected by rebound feeding. We observed comprehensive alterations immediately after protein restriction, including retarded growth, altered biochemical indices, and disturbed hormone secretion. Transcriptome profiling of the gastrocnemius muscle followed by gene ontology analyses revealed that “myogenic differentiation functions” were upregulated, while “protein catabolism” was downregulated as a compensatory mechanism, with enhanced endoplasmic reticulum stress and undesired apoptosis. Furthermore, methylome profiling of the gastrocnemius muscle showed that protein restriction altered the methylation of apoptotic and hormone secretion-related genes. Although most of the alterations were reversed after rebound feeding, 17 genes, most of which play roles during muscle development, remained altered at the transcriptional level. In summary, early-life protein restriction may undermine muscle function in the long term and affect skeletal muscle development at the both transcriptional and methylation levels, which may hazard future muscle health.

## 1. Introduction

Environmental conditions in early life can modulate the developmental programs of organisms, thereby affecting the state of health and risk of disease. These effects may originate from different events (malnutrition, exposure to infection or hazardous chemicals, and mental stress) and modulate via various mechanisms, such as epigenetic, metabolic, and endocrine mechanisms), while occurring at different stages of development, including the periconceptional, perinatal, and postnatal stages [1,2].

Malnutrition refers to the imbalance between the body’s requirement and intake of nutrients caused by both oversupply and undersupply [3]. Balanced nutrition may play an essential role in skeletal muscle development and contribute to maintaining muscle mass and quality during healthy aging [4]. On the contrary, the consequences of early life nutrition restriction, especially protein restriction, are far-reaching and influential. For instance, human aging cohort studies have shown that low birth weight due to malnutrition during pregnancy is strongly associated with decreased muscle volume and strength in later life [5,6,7]. Besides general overnutrition or undernutrition, overconsumption or deficiency of essential nutrients also stunts organ and tissue development, resulting in later-life risks [8,9]. In vivo studies have shown that early-life protein-restricted mice pups have a weak cardiovascular function, altered muscle fiber composition, and reduced exercise capacity in the adult stage [8]. Protein restriction in utero is associated with impaired structures and functions of several organs, including the liver, kidney, and pancreas [10,11,12,13]. Fetal protein malnutrition causes liver steatosis, disrupts nephrogenesis, and disturbs β-cell function and insulin secretion; therefore, leading to hyperlipidemia, hypertension, and glucose intolerance [14,15]. It has also been demonstrated that after weaning, a catch-up of dietary nutrition may reverse unwanted trends caused by maternal malnutrition [15]. These findings strongly indicate that early life weight and nutrition stimuli significantly affect skeletal muscle health in older age. However, few studies have examined the effects of protein restriction during the neonatal stages, especially on muscle development and function. In addition, the extent to which postnatal nutrition strategies may contribute to health status later in life remains to be determined.

Therefore, the present study aimed to investigate the effects of one-month protein restriction after weaning on overall growth, skeletal muscle development, function, and muscle epigenomic alterations. To investigate whether compensatory feeding is effective, we also established a subgroup of animals that received a two-month rebound feeding after protein restriction.

## 2. Results

### 2.1. Effects of Low-Protein Diets on Body Weight and Food Intake

**Figure 1:**
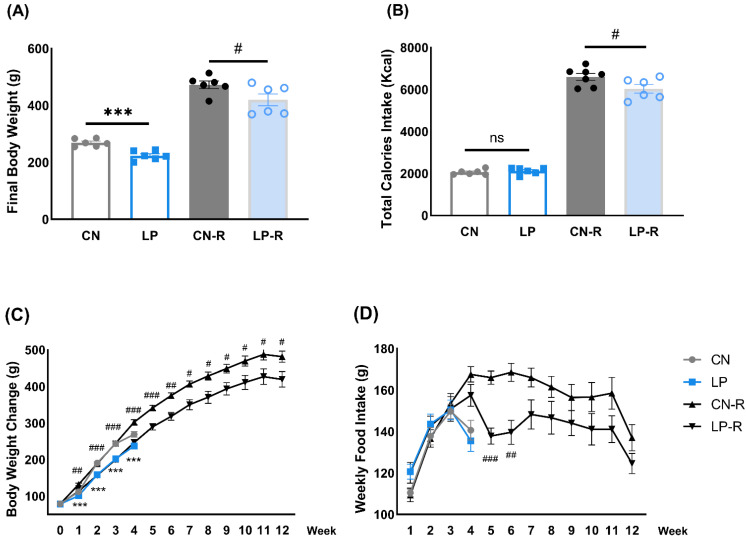
Changes in calorie intake and body weight. (**A**) Final body weight; (**B**) Total calories intake; (**C**) Weight change throughout the experiment; (**D**) Weekly food intake. Data are shown as means ± standard error of the mean (SEM), n = 6 or 7. CN, control diet (15% casein); LP, low-protein diet (8% casein); CN-R, rebound feeding group fed with control diet after 4 weeks of normal feeding; LP-R, rebound feeding group fed with control diet after 4 weeks of low-protein diet feeding; ns, no significance; *** *p* < 0.001 vs. CN. ^#^ *p* < 0.05, ^##^ *p* < 0.01, and ^###^ *p* < 0.001 vs. CN-R.

Figure 1 shows the effects of low protein diets (LP; 8% casein diet) on alterations in body weight and food intake. At the end of the 4th week, the rats fed an LP had significantly lower body weight than those fed with a 15% casein diet (CN) (Figure 1A), although they consumed similar calories (Figure 1B). The dynamic changes in body weight and food intake throughout the study are shown in Figure 1C,D. The average body weight in the LP group lagged behind that of the CN group from the 1st week to the 4th week (Figure 1C); however, no difference in food intake was observed at any measurement point (Figure 1D).

After 4 weeks of feeding with the designated diets, the LP and CN rats were fed CN for 2 months (hereinafter referred to as rebound feeding subgroups; LP-R and CN-R, respectively. For comparisons, the LP and CN groups are also referred to as regular feeding subgroups. Similar to the results of the LP and CN groups, the LP-R subgroup showed significantly lower body weight than the CN-R subgroup after the two-month rebound feeding. However, unlike the results in the regular feeding subgroup, the total calories consumed by the LP-R subgroup was significantly lower than that consumed by the CN-R subgroup. The average body weight of the LP-R subgroup was significantly lower than that of the CN-R subgroup from the 1st week to the 12th week (Figure 1C). Immediately after the LP diet was switched to the normal diet, the weekly food intake of the LP-R subgroup showed a significant decrease compared to the CN-R subgroup from the 5th to the 7th week. Afterward, a non-significant decrease was observed until the 12th week (Figure 1D).

### 2.2. Effects of Low-Protein Diets on Organ and Tissue Weight

As shown in Table 1, in the regular feeding subgroup, the absolute liver, kidney, and skeletal muscle weights of the LP rats were significantly lower than those of the CN rats. Furthermore, the kidney and skeletal muscle weights relative to body weight after fasting in the LP group (0.7% and 4.1%, respectively) were significantly lower compared to the CN group (0.8% and 1.7%, respectively; *p* < 0.05), while relative adipose tissue weight showed the opposite behavior (4.1% vs. 3.2%, respectively; *p* < 0.05). Within the muscle tissue, the relative gastrocnemius muscle weights in the LP rats was significantly lower compared to the CN rats (1.07% and 0.97, respectively; *p* < 0.05).

In the rebound feeding subgroup, although the absolute skeletal muscle weight of LP-R rats was lower than that of CN-R rats (*p* < 0.05), the absolute liver, kidney, and adipose tissue weights did not show significant changes. The weights of these organs and tissues relative to body weight after fasting also showed no difference except for the soleus muscle, which was significantly lower in the LP-R rats than that in the CN-R rats (0.10% vs. 0.09%, respectively; *p* < 0.05, Table 1).

### 2.3. Effects of Low-Protein Diets on Blood and Tissue Biochemical Parameters

As shown in Table 2, among the parameters measured, in plasma, LP rats had decreased albumin (A; *p* < 0.001) and triglyceride (TG; *p* < 0.01), and elevated glutamic oxaloacetic transaminase (GOT; *p* < 0.05) and glutamic pyruvic transaminase (GPT; *p* < 0.001) compared to CN rats. In serum, LP rats had significantly lower insulin-like growth factor 1 (IGF-1) and blood urea nitrogen (BUN) concentrations than CN rats (*p* < 0.001), leading to a significantly lower BUN/creatinine (CRE) ratio in the LP group than that in the CN group (*p* < 0.001). The hepatic total cholesterol (TC) and TG levels were significantly higher in LP rats than those in CN rats, showing the opposite trend compared to the blood counterparts. Furthermore, plasma globulin (G), glucose, GOT/GPT, non-esterified fatty acids (NEFA), TC, CRE, and gastrocnemius TG concentrations remained unaltered.

In contrast, after two months of rebound feeding, most of the altered biochemical markers in the LP-R rats recovered to a similar level as those of the CN-R rats; however, plasma A/G was significantly reduced in the LP-R group compared to that in the CN-R group.

### 2.4. Amino Acids Profile in the Plasma, Liver, or Gastrocnemius Muscle

**Figure 2:**
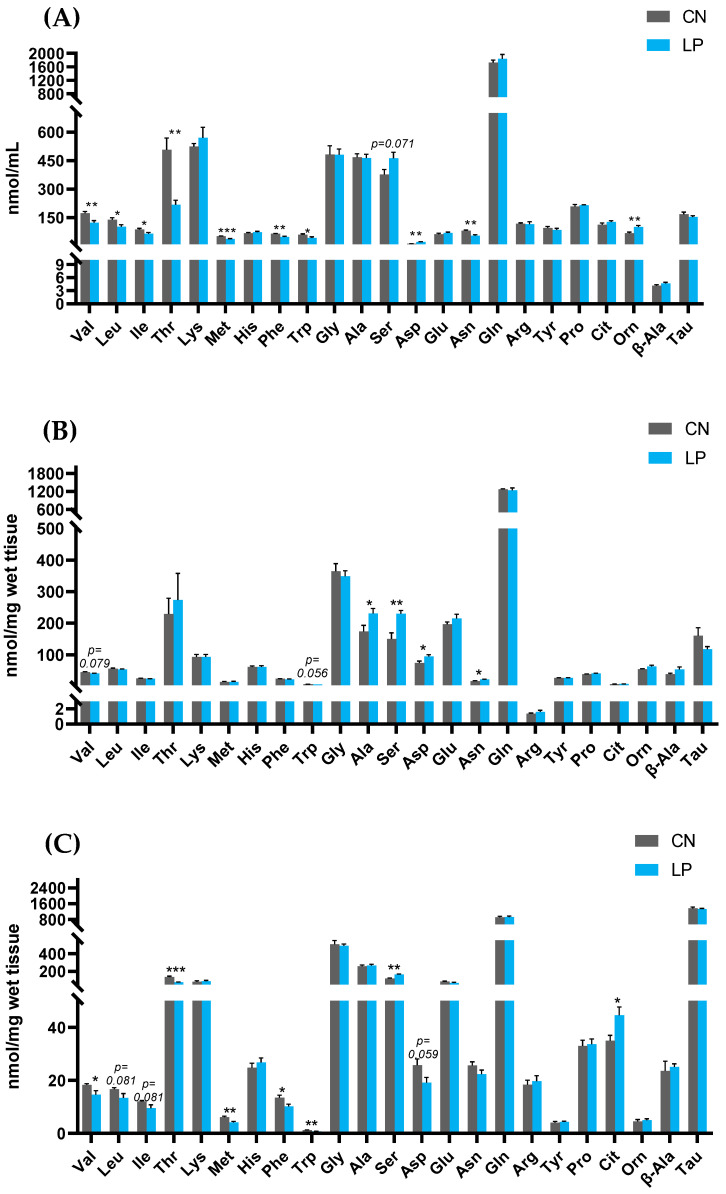

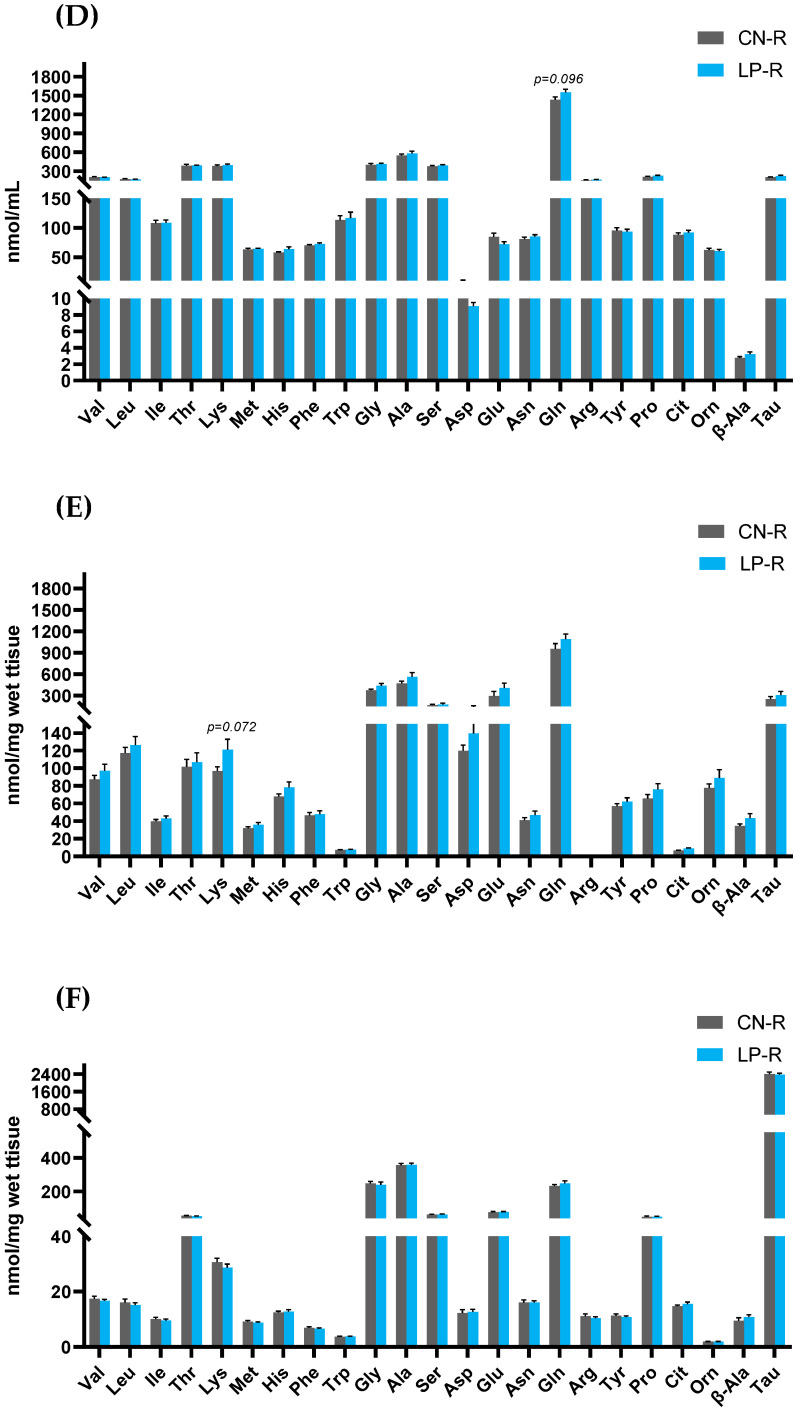
Effects of low protein diet and rebound feeding of control diet on amino acid profiles of LP and CN rats (**A–C**) during regular feeding in (**A**) plasma, (**B**) liver, and (**C**) gastrocnemius muscle; (**D–F**) during rebound feeding in (**D**) plasma, (**E**) liver, and (**F**) gastrocnemius muscle. Data are shown as means ± SEM; n = 6 or 7. CN, control diet (15% casein); LP, low-protein diet (8% casein); CN-R, rebound feeding group fed with control diet after 4 weeks of normal feeding; LP-R, re-bound feeding group fed with control diet after 4 weeks of low-protein diet feeding; * p < 0.05, ** p < 0.01, and *** p < 0.001 vs. CN. Val, valine; Leu, leucine; Ile, isoleucine; Thr, threonine; Lys, lysine; Met, methionine; His, histidine; Phe, phenylalanine; Trp, tryptophan; Gly, glycine; Ala, alanine; Ser, serine; Asp, aspartic acid; Glu, glutamic acid; Asn, asparagine; Gln, glutamine; Arg, arginine; Tyr, tyrosine; Pro, proline; Cit, citrulline; Orn, ornithine; β-Ala, β-alanine; Tau, taurine.

We measured the concentrations of both proteinogenic and non-proteinogenic amino acids. The findings revealed that protein restriction considerably lowered plasma valine (Val), leucine (Leu), isoleucine (Ile), threonine (Thr), methionine (Met), phenylalanine (Phe), tryptophan (Trp), and asparagine (Asn) levels, while it increased aspartic acid (Asp) and ornithine (Orn) levels in LP rats compared to those in CN rats (Figure 2A). The hepatic amino acid constitution was mildly affected by protein restriction compared to that in the plasma. The levels of alanine (Ala), serine (Ser), Asp, and Asn were significantly increased, and that of Val and Trp were marginally decreased in LP rats compared to those in CN rats after a 4-week protein restriction (Figure 2B). In the gastrocnemius muscle, Val, Thr, Met, Phe, and Trp concentrations were significantly lower, and the levels of Ser and citrulline (Cit) were significantly higher in the LP group than those in the CN group (Figure 2C). However, these changes in the plasma, liver, and gastrocnemius muscle of LP rats, induced by protein restriction during the regular feeding for 4 weeks, disappeared after the rebound feeding for 8 weeks (Figure 2D–F).

### 2.5. Gene Ontology (GO) and Pathway Enrichment Analyses based on Transcriptome Results in Gastrocnemius Muscle

#### 2.5.1. A Summary of Differentially Expressed Genes (DEGs) between CN and LP Groups and CN-R and LP-R Groups

DEGs in the LP or LP-R groups compared to those in the CN or CN-R groups, respectively, were extracted for further analysis. Briefly, in the LP group, 513 genes were upregulated and 492 genes were downregulated compared to the CN group. In the LP-R group, 31 genes were upregulated, and 69 genes were downregulated compared to the CN-R group.

#### 2.5.2. Functional Annotation of DEGs and GO Functional Enrichment Analysis

Biological processes related to DEGs were identified using the GO and Kyoto Encyclopedia of Genes and Genomes (KEGG) pathway analyses. The enriched categories of biological processes for the genes regulated by protein restriction are presented in Table 3, and the full list is shown in Table S1.

Functional enrichment analysis revealed alterations in several functional categories by protein restriction. The main altered categories included: apoptosis, cell growth, endothelial cell differentiation, homeostasis, mitochondrial function and cytoskeleton, protein catabolism, skeletal muscle adaptation and sarcoplasmic reticulum, skeletal muscle development, and stimulus response. Subcategories belonging to the above functional categories are subsequently extracted based on their potential roles during muscle development. In the category of apoptosis, “positive regulation of apoptotic signaling pathway” and “positive regulation of cysteine-type endopeptidase activity involved in apoptotic process” terms appeared to be elevated in the LP group, indicating an increase in myocyte apoptosis and abnormal muscle development. In the protein catabolism category, terms such as “proteasome-mediated ubiquitin-dependent protein catabolic process (UPS)” and “positive regulation of protein catabolic process” were extracted. The key genes of the UPS were downregulated in the LP group, indicating decreased protein catabolism, which could be attributed to the blunted metabolism in the LP group due to a limited protein source. In the category of skeletal muscle adaptation and sarcoplasmic reticulum, “response to denervation involved in regulation of muscle adaptation” was downregulated, indicating less neurogenesis due to reluctant protein breakdown (also known as “muscle turnover”). Detailed information on genes related to the above categories and their functions are listed in Table S2. Several genes, including SIN3 transcription regulator family member B (*Sin3b*), cysteine and glycine-rich protein 3 (*Csrp3*), gap junction protein alpha 1 (*Gja1*), early growth response protein 1 (*Egr1*), insulin-like growth factor 1 *(Igf1)*, transforming growth factor beta 2 (*Tgfb2*), SMAD Family Member 3 (*Smad3*), and NADPH Oxidase 4 (*Nox4*), all belonging to the myogenic differentiation category, were upregulated in the LP group compared to those in the CN group. On the contrary, compared to the CN group, androgen receptor (*Ar*), the important component of the IGF-1/PI3K/Akt signaling pathway that regulates mTOR activity and, therefore, affects muscle hypertrophy, was downregulated in the LP group. In addition, genes crucial for the calcium release by the sarcoplasmic reticulum process were also downregulated in the LP group, indicating decreased myocyte function in the LP group compared to that in the CN group.

After the rebound feeding period, only approximately 10% of the transcriptomic alternations were retained compared to those observed immediately after protein restriction (100 genes vs. 1005 genes). A comparison of the transcriptome results revealed a consistent impact of protein restriction in the LP and LP-R groups. As shown in Table 4, alterations in 17 genes remain unchanged after the rebound feeding (LP-R vs. LP; 6 genes remained upregulated, and 11 genes remained downregulated). Some of these genes were categorized as “muscle structure development” or “protein catabolism”, suggesting that early protein restriction has a persistent influence on muscle function.

### 2.6. DNA Methylation Status

#### 2.6.1. Overview

Promoters were defined as the regions around the transcription start sites (TSS) and were classified into three categories: (1) High-CpG-density promoter (HCP): promoters containing a 500-bp window with GC content ≥ 55% and CpG observed to an expected ratio (O/E) ≥ 0.6 within −700 to +200 bp around TSS; (2) Low-CpG-density promoter (LCP): promoters without 500-bp interval and a CpG O/E ≥ 0.4; 3); and (3) Intermediate-CpG-density promoter (ICP): remaining promoters that were neither HCP nor LCP.

Early protein restriction affected the methylation levels of 1056 methylated promoters in the gastrocnemius muscle (*p* < 0.01, M-value difference > 0.4) with an approximate 55:45 distribution of hypermethylated versus hypomethylated CpGs (589 vs. 473). The breakdown of the extracted hypermethylated genes was as follows: 350 HCP-CpGs, 138 ICP-CpGs, and 101 LCP-CpGs, while the counterpart of the extracted hypomethylated genes was 230 HCP-CpGs, 134 ICP-CpGs, and 109 LCP-CpGs.

#### 2.6.2. GO Analysis and Functional Annotation of Differentially Methylated Genes (DMGs)

DMGs were analyzed using GO and KEGG pathway analyses. As shown in Table 5, GO terms related to ATP synthesis coupled with proton transport, the regulation of peptide hormone secretion, peptide hormone secretion, and the regulation of hormone secretion ranked higher in hypermethylated promoter CpGs. However, in hypomethylated promoter CpGs, GO terms related to amino acid import across the plasma membrane and apoptosis ranked higher.

#### 2.6.3. Co-analysis with Transcriptome

The methylation of promoters negatively regulates the expression of related genes. Table 6 shows the synergism activity patterns obtained from the methylome and transcriptome studies. A total of 13 genes downregulated in transcriptome analysis were hypermethylated, whereas 14 genes that were upregulated in transcriptome analysis showed hypomethylation.

## 3. Discussion

Nutritional programming during infancy has been identified as an indicator of diseases in adulthood. Protein restriction during early life stages can result in persistent functional impairment. For example, liver or skeletal muscle mitochondrial DNA is reduced in early postnatal malnourished rats, even when proper nutrition is supplied after weaning [11]. The insulin secretory response could not be restored in adulthood if fed low-protein diets for 3 weeks after weaning [13,14]. However, the later-life genome-wide alteration with proper nutrition afterward and the existence of epi-memories of tissues and organs have not yet been clarified.

Bedi et al. (1980) investigated the lasting effects of undernutrition (simple calorie restriction; the same fodder was used across groups) on post-weaning rats [15]. From the 30th day after birth, the rats received food that could only provide approximately half of the calories they needed daily for a month, consecutively. Subsequently, they were allowed free access to food. Though the one-month calorie restriction caused a significant lag in both muscle weight and muscle/body ratio, 220 days of regular feeding reversed the growth retardation. Therefore, Bedi et al. postulated that post-weaning undernutrition could be reversed.

In this study, we designed a rebound feeding model to investigate whether the effects of after-weaning protein restriction are reversible. However, in our study, after switching to the control diet, the LP-R rats consumed significantly less food, although they were fed *ad libitum*. Immediately after switching to the normal diet, the former LP group show a sudden drop in food intake. The reason may be the high protein content in the control diet compared to the low-protein diet, which may enhance the sensation of fullness. Although our rebound feeding period was shorter than that in the study of Bedi et al. [15]., the significant body weight difference in our study may not be diminished owing to differences in dietary protein content. For organ/tissue composition, we found that a one-month protein restriction significantly interfered with growth by decreasing the muscle/body weight % while increasing adiposity. The two-month compensating feeding reversed these changes in muscle/adipose tissue composition. For biochemical parameters, one-month protein restriction decreased plasma albumin and TG levels, blood and tissue amino acid profiles, increased hepatic lipid accumulation, and induced a parasecretion of IGF-1, which seemed to be transient. Although these changes were reversed by the two-month rebound feeding, the situation may change if a second burden, such as a high-fat diet, is received. Previously, we reported that offspring that have undergone maternal protein restriction are more vulnerable to diet-induced obesity, while plasma amino acid profiles fluctuate more in maternal undernourished offspring [16]. Here, we show that afterbirth protein restriction affects the amino acid profile; however, these fluctuations seem reversible.

In the regular feeding subgroup, we observed a significant downregulation or upregulation in the expression of genes and pathways related to skeletal muscle adaptation and sarcoplasmic reticulum, protein catabolism, or apoptosis, respectively. In the protein catabolism category, we identified various genes related to protein degradation, including 26s proteasome, ubiquitin ligases, and endoplasmic reticulum-associated degradation (ERAD), downregulated by post-weaning protein restriction. This could be attributed to an overall decrease in protein turnover due to dietary protein deficiency. Muscle protein breakdown is an important metabolic component of muscle development and remodeling, and an inactive state in developing muscles may reduce the ability to increase muscle mass [17]. Moreover, functional enrichment analysis showed that the forkhead box O (FoxO) signaling pathway was also inhibited. It has been reported that protein restriction deactivates FoxO signaling, thus inhibiting muscle RING-finger protein 1 (MuRF1) and Atrogin-1, both of which are affiliated with atrogenes [18]. Though genes encoding MuRF1 were not altered by protein restriction, F-box protein 32 (*fbxo32*), which encodes atrogin-1, increased by >1.9 fold in the LP group, according to our results. Our results also indicated that inactivated protein degradation followed by protein restriction may originate from the inhibition of the FoxO signaling pathway. ERAD is a process in which proteins in the endoplasmic reticulum (ER) are dislocated to the cytosol for proteasomal degradation, an important response caused by unfolded or incorrectly folded proteins in the secretory pathway [19]. The downregulated ERAD signal may originate similarly to FoxO, leaving misfolded proteins unprocessed, thus increasing ER stress and further prompting apoptosis [19,20].

In the category of apoptosis, we found that genes encoding inducing factors of apoptosis, such as tumor necrosis factor (TNF) superfamily member 10 (*Tnfsf10*), TNF receptor superfamily member 12A (*Tnfrsf12a*), caveolin 1 (*Cav1*), and cellular communication network factor (*Cyr61*), which have been reported in previous studies for their apoptotic gene encoding functions, were upregulated [20,21,22]. Meanwhile, activating transcription factor 3 (*ATF3*), a transcription factor of the TNF-related apoptosis-inducing ligand (TRAIL) receptor, was also upregulated [23]. Previous studies have reported that the apoptotic process is enhanced in denervated, aging, or disused muscles [24,25]. Our results indicate that early-age protein restriction may harm muscle development by aggravating unwanted apoptosis.

In the category of skeletal muscle adaptation and sarcoplasmic reticulum, alternations of subcategories such as the response to denervation involved in regulation of muscle adaptation and the regulation of release of sequestered calcium ion into cytosol by sarcoplasmic reticulum were recognized. The altered profile of genes belongs to the term protein catabolism, which we have already pointed out, might be attributed to an accompanying phenomenon of atrophy induced by a denervation-like behavior generated by protein restriction [26]. Meanwhile, the abnormal calcium ion release may bring functional dissonance during muscle contraction and relaxation [27].

In the comparison between the LP and LP-R groups, we found a marked decrease in DEG counts. However, *Cyr61*, also known as CCN1, is responsible for wound healing/muscle regeneration, [28] and palladin, a cytoskeletal-associated protein (*Palld*), both of which are related to myogenic differentiation, remained upregulated. These phenomena are associated with muscle hyperdifferentiation and muscle satellite cell depletion and impair muscle repairing capacity. Moreover, *palld* polymorphism is involved with MyoD and Myogenin protein production in C2C12 cells [29]. In contrast, the cell phase-regulating gene cyclin-dependent kinase inhibitor 1A (*Cdkn1a*) remained downregulated, indicating over-proliferation and differentiation in satellite cells, thus undermining muscle function [30,31,32]. Furthermore, nuclear receptor subfamily 4 group A members 1 and 2 (*Nr4a2* and *Nr4a1*), regulators of glucose metabolism and mitochondrial oxidation, were downregulated, indicating an adverse impact on skeletal muscle homeostasis [33]. We also found that CCAAT enhancer binding protein delta (*Cebpd*) and phosphodiesterase 4B (*Pde4b*), which inversely correlate with inhibition to muscle atrophy, showed lasting effects in the LP and LP-R subjects [34,35]. Therefore, further studies are needed to confirm the interplays between those antagonizing effects of the regulated genes.

We identified a variety of differentially expressed methylated genes between CN and LP groups. Previous studies involving protein restriction and methylome profiling have focused on the impact of maternal protein restriction on mothers and offspring [36,37,38], while our study is the first to reveal how post-weaning protein restriction affects the offspring methylome profile.

Functional enrichment analyses of the methylome indicated that post-weaning protein restriction induced: (1) hypermethylation of ATP synthesis-related genes, (2) hypermethylation of peptide hormone secretion-related genes, and (3) hypomethylation of apoptotic genes. Previous studies have reported that calorie restriction, including protein restriction, favors mitochondrial biogenesis efficiency; however, our results indicate that early protein restriction may induce dysfunction in muscle mitochondria and ATP synthesis abnormalities, indicating that DNA methylation may be a potential reason [39,40]. The results of hypomethylation of apoptotic genes correlate with the transcriptome results and a previous report that hyperhomocysteinemia, a consequence of hypermethylation, may induce myocyte apoptosis through oxidation [41]. In addition, the altered “amino acid import across the plasma membrane” category is also a plausible reason for the abnormal amino acid profiles in LP rats. These results indicate that methylation may be one of the mechanisms by which the transcriptome is altered. Co-analysis of the transcriptome and methylome data revealed that out of 513 genes upregulated in transcriptome analysis, 14 genes were hypomethylated, while 13 of the 492 downregulated genes were hypermethylated. These results indicate that post-weaning protein restriction regulates these genes at the DNA methylation and transcriptional levels, consistent with several previous reports [42,43].

However, when comparing the transcriptome profiles of LP and LP-R groups, we did not observe a lasting effect on any of these 27 genes. These results suggest that a two-month rebound feeding apparently washed out the expressional alterations caused by protein restriction-induced methylation. Previous studies have reported that early life nutrient stress may help form an “epi” memory in the programmable skeletal muscles [43,44]. In other words, if the stimuli experienced in early life are re-encountered in later life, the “epi” memory will help confront the same dilemma with a quicker response. We adopted a relatively short experiment period with only rebound feeding in this study. Moreover, we investigated methylation status at only one timepoint, and may have overlooked some of the changes in the methylating states in the previous or following periods. Since the methylation memory is usually more persistent than the transcriptional status, it may be informative to investigate the differential methylation patterns at different recovery timepoints. Therefore, further studies are required to investigate the long-term effects of post-weaning protein restriction evaluated over a series of time points. At the same time, a second-time protein restriction stimulus should also be introduced to investigate to what extent an “epi” memory will be constituted by post-weaning protein restriction. Another limitation of this study is that we did not implement protein-level analyses, muscular functional test, such as a four-limb grip power test or exercise tolerance test, or perform histochemical observations to evaluate the fiber type composition, muscle cell area, or muscle satellite cell distribution. Further studies are necessary to investigate the phenotypic changes based on our current results. In addition, mitochondrial biogenesis, mitophagy, and structural network organization play vital roles during muscle development and regeneration. The present genome-wide analyses revealed that post-weaning protein restriction may induce transient and long-lasting effects on mitochondrial function; however, further studies focusing on this topic are encouraged, based on our current results.

Taken together, our present results indicated that post-weaning protein restriction affects skeletal muscle development at both the transcriptional and methylation levels. A two-month rebound feeding may reverse most of the dilemma by recovering methylated status; however, transcriptional alteration continuously exists. Our current results indicate that rapid remedial nutrition measures may ameliorate adverse effects caused by post-weaning undernutrition to some extent; nevertheless, growth latency and other delayed impacts cannot be ignored.

## 4. Materials and Methods

### 4.1. Animals and Diets

**Figure 3:**
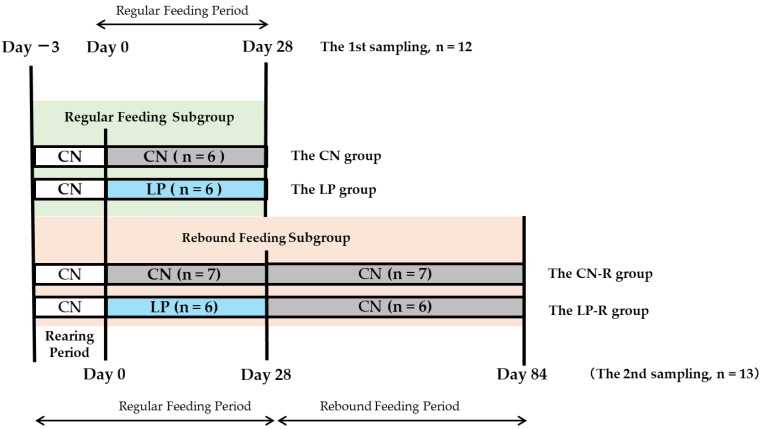
Study design. CN: control diet; LP: low-protein diet. CN-R, CN followed by rebound feeding. LP-R, LP followed by rebound feeding.

Male Wistar rats (3.5-week-old) were purchased from Charles River Japan (Kanagawa, Japan) and kept in a room maintained at 22 ± 2 °C with a relative humidity of 40–60% and a 12 h light (8:00–20:00) and 12 h darkness (20:00–8:00) cycle. The animals were allowed free access to water and food throughout the experiments. The study began when the rats were 4 weeks old. After the rats were fed a purified diet containing 15% casein protein for three days, they were randomly divided into two groups with the same average weight. Rats in the first group (n = 13) were provided with a 15% casein diet (CN) as a control diet, and those in the second group (n = 12) were fed a calorie-matched 8% casein diet (LP), as a low-protein diet for 4 weeks (Figure 3). Nutritional information on the diets is shown in Table S3. On the 27th day, the rats in each group were divided into two subgroups: CN and CN-R and LP and LP-R (n = 6 in CN, LP, LP-R, n = 7 in CN–R.). For the CN and LP subgroups, the rats were anesthetized with isoflurane (Fujifilm Wako Pure Chemical Corp., Osaka, Japan) after a 16-h fasting. The rats in CN-R and LP-R subgroups were kept on the control diet, and on the 84th day, sampling was conducted after a 16-h fast. After sacrifice, blood samples were collected from the carotid artery. The liver, kidney, muscles, and abdominal fat pads were harvested and weighed. Plasma or serum was separated from the blood, while the organs and tissues were soaked in RNAlater solutions (AMBION, Austin, TX, USA) or snap-frozen in liquid nitrogen and stored at −80 °C until use.

### 4.2. Biochemical Tests and Amino Acid Profiling

Blood biochemical parameters and amino acid profiles were measured according to the manufacturer’s protocol. Briefly, blood biochemical parameters, including plasma albumin, TC, glucose concentrations, plasma or serum GOT and GPT, NEFA, and hepatic and muscular TG levels, were determined using commercial kits (Cholesterol E-test, Triglyceride E-test, NEFA C-test, and Glucose C II test, respectively, Fujifilm Wako Pure Chemical Industries). For lipid content in the liver or muscle tissue, lipids were extracted from frozen livers and gastrocnemius muscles via the modified Folch method in a 2:1 (vol/vol) mixture of chloroform/methanol. The extracts were washed with 0.5 volume of 0.8% KCl and centrifuged at 1500× *g* for 10 min, and the organic phases were recovered.

Serum BUN, CRE and IGF-1 concentrations were measured using UN-S assay kit (Denka, Tokyo, Japan), L-type Wako CRE-M (Wako), and Quantikine^®^ ELISA kit (Mouse/Rat ELISA Kit MG-100, R&D systems, Minneapolis, MN, USA), respectively.

To analyze amino acid in the plasma, liver, and gastrocnemius muscle, plasma samples were mixed with 2 volume acetonitrile and 1 volume deionized water, then centrifuged at 14,500 rpm for 2 min at 4 °C, and supernatants were collected. The liver and gastrocnemius muscle samples (100 mg) were homogenized with 1 mL acetonitrile and 0.5 mL deionized water, then centrifuged at 1100 rpm for 3 min at 4 °C and supernatants were collected. Subsequently, the supernatants of plasma, liver, and gastrocnemius muscle samples were filtered through a 0.2 µm membrane and used for the analysis. The amino acid analysis was performed using a NexeraX2 automatic amino acid analyzer (Shimadzu, Kyoto, Japan).

### 4.3. RNA and DNA Extraction

Total RNA was isolated from homogenized gastrocnemius muscle using the RNeasy Fibrous Tissue Mini Kit (Qiagen, Hilden, Germany) according to the manufacturer’s instructions. The total RNA concentration was measured using a NanoDrop^®^ spectrophotometer (ND-1000, NanoDrop, Wilmington, DE, USA). The quality of the RNA was determined by assessing the A260/280 and A260/230 ratios and agarose gel electrophoresis.

Total DNA was isolated from homogenized gastrocnemius muscle using DNA iso reagent (Takara Bio, Shiga, Japan). Isolated DNA was dissolved in 0.5 mL Tris-EDTA buffer (Nippon Gene, Toyama, Japan). After ribonuclease digestion using RNase A (Takara Bio), ethanol precipitation was performed following the manufacturer’s instructions, and DNA was obtained. The quality of the DNA was determined by assessing the A260/280 and A260/230 ratios and agarose gel electrophoresis.

### 4.4. DNA Microarray and Functional Enrichment Analyses

Four individuals (one from each treatment group) whose body weight was similar to the average weight of the respective group were selected and the gastrocnemius muscle was used for analyses. DNA microarray analysis was performed using a Genechip^®^ Rat Genome 230 2.0 Array (Thermo Fisher Scientific, Waltham, MA, USA). Data were normalized by R [45] using a Factor Analysis for Robust Microarray Summarization (FARMS) [46], and the DEGs between the two groups were identified using the rank products 2.0 method [47]. The probesets were filtered under the condition of a false discovery rate ≤0.05, and their functions were analyzed using Metascape 3.5 (https://metascape.org/gp/index.html#/main/step1, accessed on 1 July 2020) [48]. Raw data generated from scans were normalized using log_2_ ratio. Genes with a log_2_ (*q*-value) < −2 were extracted and analyzed using GO terms [49] and KEGG pathway analyses. The identified GO terms were confirmed for GO annotations using Quick GO (https://www.ebi.ac.uk/QuickGO/annotations, accessed on 1 July 2020) [50].

### 4.5. Differentially Methylated Region Identification, Promoter Classifications, and Functional Enrichment Analysis

Three individuals (one from each treatment group) whose body weights were similar to the average weight of the respective group were selected and their gastrocnemius muscles used for analyses. Differences in methylation were studied using a combination of genome-wide DNA methylation arrays and methylated DNA immunoprecipitation at Arraystar Inc. (Rockville, MD, USA) by methylated DNA immunoprecipitation (MEDIP). For DNA labelling, the NimbleGen Dual-Color DNA Labeling Kit was used according to the manufacturer’s guideline detailed in the NimbleGen MeDIP-chip protocol (Nimblegen Systems, Inc., Madison, WI, USA). Briefly, 1 μg DNA of each sample was incubated for 10 min at 98 °C with 1 OD of Cy5-9mer primer (IP sample) or Cy3-9mer primer (Input sample). Then, 100 pmol of deoxynucleoside triphosphates and 100U of the Klenow fragment (New England Biolabs, Ipswich, MA, USA) were added and the mix was incubated at 37 °C for 2 h. The reaction was stopped by adding 0.1 mL of 0.5 M EDTA, and the labeled DNA was purified by isopropanol/ethanol precipitation. Raw data generated from scans were normalized (log_2_ ratio). We performed median centering, quantile normalization, and linear smoothing using Bioconductor packages Ringo, limma, and MEDME. The normalized data were further analyzed using a sliding-window peak-finding algorithm provided by NimbleScan v2.5 (Roche-NimbleGen, Madison, WI, USA) to find the enriched peaks with specified parameters (sliding window width 1500 bp; minimum probes per peak 2; *p*-value minimum cut-off (−log_10_) 2), and peaks within 500 bp spacing were merged.

To compare the differentially enriched regions between the two groups, the log_2_ (MeDIP/Input) was averaged for each group, and the M’ value for each probe was calculated as follows: M’ = Average (log_2_MeDIPE/InputE) − Average (log_2_MeDIPC/InputC). Then, the NimbleScan sliding-window peak-finding algorithm was rerun on these data to find the differential enrichment peaks (DEPs) between groups, filtered according to the following criteria: (1) At least one of the two groups had a median (log_2_ MeDIP/Input) ≥ 0.3 and a median (M″) > 0; (2) At least half of the probes in a peak had a coefficient of variability ≤ 0.8 in both groups.

The identified gene, whose promoters showing significant alterations in DNA methylation were further characterized by GO functional enrichment analyses.

### 4.6. Statistical Analysis

Results are expressed as the mean ± standard error of the mean (SEM). Multiple group comparisons were performed using one-way analysis of variance followed by Tukey’s test using the GraphPad Prism software (GraphPad Prism 9.0, San Diego, CA, USA). A *p*-value of <0.05 was considered significant.

## Figures and Tables

**Table 1:** Absolute or relative organ/tissue weight at the end of the studies.

Organ or Tissue	CN	LP	CN-R	LP-R
Liver, g	8.1 ± 0.4	6.9 ± 0.3 *	11.94 ± 3.1	10.3 ± 0.7
Kidney, g	2.1 ± 0.1	1.5 ± 0.1 ***	2.5 ± 0.1	2.4 ± 0.1
Total adipose tissue, g	8.6 ± 0.7	9.2 ± 0.7	35.9 ± 2.1	29.7 ± 3.6
Retroperitoneal fat pad	3.2 ± 0.3	3.6 ± 0.4	13.2 ± 1.5	12.5 ± 1.7
Mesenteric fat pad	1.9 ± 0.2	2.2 ± 0.2	8.3 ± 0.9	7.9 ± 0.9
Epididymal fat pad	3.4 ± 0.2	3.4 ± 0.2	9.6 ± 1.1	9.3 ± 1.1
Total skeletal muscle tissue, g	5.1 ± 0.1	3.9 ± 0.2 ***	9.5 ± 0.3	8.2 ± 0.4 #
Gastrocnemius muscle	2.9 ± 0.0	2.2 ± 0.1 ***	5.3 ± 0.3	4.7 ± 0.3 #
Soleus muscle	0.3 ± 0.0	0.2 ± 0.1 **	0.5 ± 0.0	0.4 ± 0.0 ##
Plantaris muscle	0.6 ± 0.0	0.4 ± 0.0 ***	1.1 ± 0.1	1.0 ± 0.0 #
Extensor digitorum longus muscle	0.3 ± 0.0	0.2 ± 0.0 ***	0.5 ± 0.0	0.4 ± 0.0 ##
Tibialis anterior muscle	1.1 ± 0.0	0.8 ± 0.0 ***	1.9 ± 0.1	1.7 ± 0.2 #
BW-af, g	269.3 ± 5.3	223.7 ± 6.7 ***	481.3 ± 14.3	419.5 ± 20.9 #
Liver/BW-af, %	3.0 ± 0.1	3.1 ± 0.0	2.5 ± 0.1	2.4 ± 0.1
Kidney/BW-af, %	0.8 ± 0.0	0.7 ± 0.0 *	0.6 ± 0.0	0.6 ± 0.0
Total adipose/BW-af, %	3.2 ± 0.2	4.1 ± 0.3 *	6.8 ± 0.6	7.0 ± 0.6
Total skeletal Muscle/BW-af, %	1.9 ± 0.0	1.7 ± 0.0 *	2.0 ± 0.1	2.0 ± 0.1
Gastrocnemius muscle/BW-af, %	1.07 ± 0.02	0.97 ± 0.03 *	1.14 ± 0.01	1.13 ± 0.04
Soleus muscle/BW-af, %	0.10 ± 0.01	0.09 ± 0.00	0.10 ± 0.00	0.09 ± 0.00 #
Plantaris muscle/BW-af, %	0.21 ± 0.01	0.19 ± 0.00	0.24 ± 0.07	0.24 ± 0.06
Extensor digitorum longus muscle/BW-af, %	0.10 ± 0.00	0.08 ± 0.00	0.10 ± 0.00	0.10 ± 0.00
Tibialis anterior muscle/BW-af, %	0.42 ± 0.01	0.40 ± 0.01	0.41 ± 0.01	0.40 ± 0.01

Data are shown as the mean ± SEM (n = 6–7); CN, control diet-fed group; LP, low-protein diet-fed group; CN-R, rebound feeding group fed with control diet after 4 weeks of normal feeding; LP-R, rebound feeding group fed with control diet after 4 weeks of low-protein diet feeding; BW, body weight; af, after fasting. * p < 0.05 and *** p < 0.001 vs. CN; *# p* < 0.05 and #*# p* < 0.01 vs. CN-R.

**Table 2:** Biochemical parameters in the plasma, serum, liver, or gastrocnemius muscle.

Parameters	CN	LP	CN-R	LP-R
Plasma, albumin (mg/dL)	3.91 ± 0.09	3.46 ± 0.06 ***	3.91 ± 0.06	3.82 ± 0.09
Plasma, globulin (mg/dL)	1.44 ± 0.06	1.38 ± 0.10	1.69 ± 0.06	1.87 ± 0.08
Plasma, A/G	2.73 ± 0.11	2.57 ± 0.17	2.33 ± 0.09	2.05 ± 0.06 #
Plasma, glucose (mg/dL)	104.1 ± 6.1	115.8 ± 12.5	185.2 ± 13.7	172.2 ± 10.8
Plasma, GOT (Karmen)	92.6 ± 4.6	118.6 ± 6.6 *		
Plasma, GPT (Karmen)	12.3 ± 0.9	17.7 ± 0.9 **		
Plasma, GOT/GPT	7.6 ± 0.3	7.7 ± 0.9		
Plasma, NEFA (mg/dL)	1.23 ± 0.07	1.12 ± 0.07	0.67 ± 0.04	0.77 ± 0.07
Plasma, TC (mg/dL)	61.6 ± 4.0	64.9 ± 4.1	59.3 ± 2.6	57.7 ± 5.2
Plasma, TG (mg/dL)	80.2 ± 3.1	55.4 ± 4.4 **	90.6 ± 15.6	76.4 ± 11.5
Serum, IGF-1 (pg/mL)	461.3 ± 22.9	256.2 ± 19.4 ***	650.2 ± 34.9	640.6 ± 37.6
Serum, BUN (mg/dL)	16.9 ± 1.0	8.3 ± 0.8 ***	11.3 ± 0.4	12.1 ± 0.5
Serum, CRE (mg/dL)	0.4 ± 0.0	0.4 ± 0.0	0.3 ± 0.0	0.3 ± 0.0
Serum, BUN/CRE	44.5 ± 2.0	21.1 ± 1.6 ***	36.0 ± 1.3	39.7 ± 1.8
Serum, GOT (Karmen)			90.1 ± 8.6	79.6 ± 3.0
Serum, GPT (Karmen)			17.4 ± 0.9	15.3 ± 0.9
Serum, GOT/GPT			5.3 ± 0.3	5.3 ± 0.3
Liver, TC (mg/g)	9.0 ± 0.7	13.2 ± 1.5 *	10.0 ± 0.4	8.9 ± 0.4
Liver, TG (mg/g)	29.8 ± 2.6	86.5 ± 14.7 **	41.5 ± 4.5	31.5 ± 3.2
Gastrocnemius, TG (mg/g)	3.7 ± 0.3	4.4 ± 0.4	6.7 ± 0.9	5.2 ± 0.5

Data are shown as the mean ± SEM (n = 6–7); CN, control diet-fed group; LP, low-protein diet-fed group; CN-R, rebound feeding group fed with control diet after 4 weeks of normal feeding; LP-R, rebound feeding group fed with control diet after 4 weeks of low-protein diet feeding; A/G, albumin/globulin; GOT, glutamic-oxaloacetic transaminase; GPT, glutamic-pyruvic transaminase; NEFA, non-esterified fatty acid; TC, total cholesterol; TG, triglyceride; IGF-1, insulin-like growth factor-1; BUN, blood urea nitrogen; CRE, creatinine; * p < 0.05, ** p < 0.01, and *** p < 0.001 vs. CN; *# p* < 0.05 vs. CN-R.

**Table 3:** Highlighted functional annotation within gene ontology (GO) terms (Transcriptome results). Up/Down arrows show an upregulating trend/downregulating trend after categorizing genes those have increased fold changes of >1.5 or decreased fold changes of <1.5 into GO function enrichment analyses. Comparison was carried out using Low protein vs. Control diet groups.

Term		Log_2_ (*q*-value)	Up/Down
Skeletal muscle development			↑
GO:0014706	striated muscle tissue development	−2.92	
GO:0007517	muscle organ development	−2.27	
Apoptosis			
GO:2001235	positive regulation of apoptotic signaling pathway	−2.58	
GO:0043280	positive regulation of cysteine-type endopeptidase activity involved in apoptotic process	−2.27	
GO:0097191	extrinsic apoptotic signaling pathway	−2.24	
Endothelial cell differentiation			
GO:0001944	vasculature development	−8.84	
GO:0045766	positive regulation of angiogenesis	−3.60	
GO:2000181	negative regulation of blood vessel morphogenesis	−2.65	
GO:0001938	positive regulation of endothelial cell proliferation	−2.78	
GO:0001937	negative regulation of endothelial cell proliferation	−2.27	
GO:0061028	establishment of endothelial barrier	−2.34	
GO:0048660	regulation of smooth muscle cell proliferation	−2.37	
GO:1904705	regulation of vascular smooth muscle cell proliferation	−2.34	
GO:0014910	regulation of smooth muscle cell migration	−2.27	
Mitochondrial function and cytoskeleton			
GO:0007005	mitochondrion organization	−8.12	
GO:0010821	regulation of mitochondrion organization	−2.08	
GO:1902903	regulation of supramolecular fiber organization	−2.05	
GO:0007015	actin filament organization	−2.15	
Stimulus response			
GO:0097237	cellular response to toxic substance	−2.91	
GO:0001666	response to hypoxia	−4.85	
GO:0002931	response to ischemia	−2.56	
GO:0009612	response to mechanical stimulus	−2.26	
Homeostasis			
GO:0106106	cold-induced thermogenesis	−2.41	
GO:0120161	regulation of cold-induced thermogenesis	−2.41	
GO:0055080	cation homeostasis	−2.01	
Others			
GO:0051345	positive regulation of hydrolase activity	−3.20	
GO:0097201	negative regulation of transcription from RNA polymerase II promoter in response to stress	−2.47	
GO:0019693	ribose phosphate metabolic process	−2.07	
GO:0045785	positive regulation of cell adhesion	−2.33	
GO:0071900	regulation of protein serine/threonine kinase activity	−2.15	
Skeletal muscle adaptation and sarcoplasmic reticulum			↓
GO:0014894	response to denervation involved in regulation of muscle adaptation	−2.60	
GO:0014888	striated muscle adaptation	−2.25	
GO:0014741	negative regulation of muscle hypertrophy	−2.64	
GO:0010880	regulation of release of sequestered calcium ion into cytosol by sarcoplasmic reticulum	−3.07	
Protein catabolism			
GO:0043161	proteasome-mediated ubiquitin-dependent protein catabolic process	−2.60	
GO:0045732	positive regulation of protein catabolic process	−2.76	
Cell growth			
GO:0071363	cellular response to growth factor stimulus	−3.24	
GO:0071383	cellular response to steroid hormone stimulus	−2.81	
GO:0016049	cell growth	−2.03	
Others			
GO:0017148	negative regulation of translation	−3.13	
GO:0015671	oxygen transport	−3.17	
GO:0034249	negative regulation of cellular amide metabolic process	−2.67	
GO:0098754	detoxification	−2.31	
GO:0045444	fat cell differentiation	−2.17	

**Table 4:** Genes that are altered in LP and LP-R rats (Transcriptome results).

Gene Symbol	Gene Title
Upregulated continuously both in LP and LP-R	
*Cyr61*	Cysteine-rich, angiogenic inducer, 61
*LOC102553868*	Uncharacterized LOC102553868
*LOC102554740/LOC103693750*	Uncharacterized LOC102554740 uncharacterized LOC103693750
*Nmrk2*	Nicotinamide riboside kinase 2
*Palld*	Palladin, cytoskeletal associated protein
*Ubap1l*	Ubiquitin-associated protein 1-like
Downregulated continuously both in LP and LP-R	
*Cdkn1a*	Cyclin-dependent kinase inhibitor 1A
*Cebpd*	CCAAT/enhancer binding protein (C/EBP), delta
*Crem*	Camp responsive element modulator
*LOC103689947/Selenbp1*	Selenium-binding protein 1
*Maf*	V-maf avian musculoaponeurotic fibrosarcoma oncogene homolog
*Nr4a1*	Nuclear receptor subfamily 4, group A, member 1
*Nr4a2*	Nuclear receptor subfamily 4, group A, member 2
*Pde4b*	Phosphodiesterase 4B, camp specific
*Retsat*	Retinol saturase (all trans retinol 13,14 reductase)
*Slc25a25*	Solute carrier family 25 (mitochondrial carrier, phosphate carrier), member 25
*Tmem100*	Transmembrane protein 100

LP, low-protein diet-fed group; LP-R, rebound feeding group fed with control diet after 4 weeks of low-protein diet feeding.

**Table 5:** Highlighted functional annotation within GO terms (Methylome results).

Term	Description	Log_2_ (*q*-Value)	Up/Down
GO:0015986	ATP synthesis coupled proton transport	−4.19	↑
GO:0030072	Peptide hormone secretion	−4.19	
GO:0046883	Regulation of hormone secretion	−4.03	
GO:0090276	Regulation of peptide hormone secretion	−4.39	
GO:0089718	Amino acid import across plasma membrane	−2.42	↓
rno04210	Apoptosis	−2.79	

**Table 6:** Specified genes within the highlighted GO terms (Transcriptome and methylome results).

Gene Symbol	Gene Title	Promoter Classification	Peak DMvalue	Transcription Status	Methylation Status
*Aff4*	AF4/FMR2 family, member 4	HCP	0.30	↓	↑
*Cd24*	CD24 molecule	HCP	0.18		
*Ctsl*	Cathepsin L	HCP	0.16		
*Maf*	V-maf avian musculoaponeurotic fibrosarcoma oncogene homolog	HCP	0.43		
*Zfp91*	Zinc finger protein 91	HCP	0.36		
*Zfyve9*	Zinc finger, FYVE domain containing 9	HCP	0.17		
*Zranb2*	Zinc finger, RAN-binding domain containing 2	HCP	0.31		
*Pdk4*	Pyruvate dehydrogenase kinase, isozyme 4	ICP	0.03		
*Prkag3*	Protein kinase, AMP-activated, gamma 3 non-catalytic subunit	ICP	0.40		
*Prkar2a*	Protein kinase, cAMP-dependent regulatory, type II alpha	ICP	0.35		
*Psmd7*	Proteasome 26S subunit, non-ATPase 7	ICP	0.30		
*Usp7*	Ubiquitin-specific peptidase 7	ICP	0.41		
*Gsta1*	Glutathione S-transferase alpha 1	LCP	0.39		
*Coa3*	Cytochrome C oxidase assembly factor 3	HCP	0.14	↑	↓
*Kcnc1*	Potassium channel, voltage-gated Shaw-related subfamily C, member 1	HCP	0.10		
*Kitlg*	KIT ligand	HCP	0.13		
*Plk2*	Polo-like kinase 2	HCP	0.45		
*Stx4*	Syntaxin 4	HCP	0.17		
*Cxcr4*	Chemokine (C-X-C motif) receptor 4	ICP	0.36		
*Jph2*	Junctophilin 2	ICP	0.14		
*Rgs4*	Regulator of G-protein signaling 4	ICP	0.21		
*Rps6ka2*	Ribosomal protein S6 kinase polypeptide 2	ICP	0.17		
*Fsd2*	Fibronectin type III and SPRY domain containing 2	LCP	0.16		
*Gpihbp1*	Glycosylphosphatidylinositol anchored high-density lipoprotein binding protein 1	LCP	0.18		
*Steap4*	STEAP family member 4	LCP	0.16		
*Tie1*	Tyrosine kinase with immunoglobulin-like and EGF-like domains 1	LCP	0.26		
*Tmem176b*	Transmembrane protein 176B	LCP	0.04		

HCP, High-CpG-density promoter; LCP, Low-CpG-density promoter; ICP, Intermediate-CpG-density promoter.

## Data Availability

All microarray data are MIAME compliant and have been deposited in a MIAME compliant database, the National Center for Biotechnology Information (NCBI) Gene Expression Omnibus (http://www.ncbi.nlm.nih.gov/geo/, accessed on 2 September 2022, GEO Series accession number GSE211928, https://www.ncbi.nlm.nih.gov/geo/query/acc.cgi?acc=GSE211928), as detailed on the FGED Society website (http://fged.org/projects/miame/, accessed on 1 October 2022).

## Supplementary Materials

The supporting information can be downloaded at: https://www.mdpi.com/article/10.3390/ijms232415771/s1.
